# Syntactic modulation of rhythm in Australian pied butcherbird song

**DOI:** 10.1098/rsos.220704

**Published:** 2022-09-28

**Authors:** Jeffrey Xing, Tim Sainburg, Hollis Taylor, Timothy Q. Gentner

**Affiliations:** ^1^ Department of Psychology, University of California San Diego, La Jolla, CA, USA; ^2^ Neurobiology Section, Division of Biological Sciences, University of California San Diego, La Jolla, CA, USA; ^3^ Kavli Institute for Brain and Mind, University of California San Diego, La Jolla, CA, USA; ^4^ Sydney Conservatorium of Music, University of Sydney, Sydney, New South Wales, Australia

**Keywords:** animal communication, birdsong, song complexity, Australian pied butcherbird, syntax, rhythm

## Abstract

The acoustic structure of birdsong is spectrally and temporally complex. Temporal complexity is often investigated in a syntactic framework focusing on the statistical features of symbolic song sequences. Alternatively, temporal patterns can be investigated in a rhythmic framework that focuses on the relative timing between song elements. Here, we investigate the merits of combining both frameworks by integrating syntactic and rhythmic analyses of Australian pied butcherbird (*Cracticus nigrogularis*) songs, which exhibit organized syntax and diverse rhythms. We show that rhythms of the pied butcherbird song bouts in our sample are categorically organized and predictable by the song’s first-order sequential syntax. These song rhythms remain categorically distributed and strongly associated with the first-order sequential syntax even after controlling for variance in note length, suggesting that the silent intervals between notes induce a rhythmic structure on note sequences. We discuss the implication of syntactic–rhythmic relations as a relevant feature of song complexity with respect to signals such as human speech and music, and advocate for a broader conception of song complexity that takes into account syntax, rhythm, and their interaction with other acoustic and perceptual features.

## 1. Introduction

Birdsong has a wide range of communicative functions [1–3], which are often thought to be mediated by acoustic complexity [1,4–7]. Predominantly, song complexity is investigated in a syntactic framework, where the song is segmented into a sequence of discrete units [8,9]. In this approach, song complexity is defined by the statistical features of the sequence, such as the number of unique units (repertoire size [1]), transition probabilities of units (sequential syntax [7]) or statistics derived from information theory (e.g. normalized entropy [10], mutual information [11,12]). Birdsong is typically characterized by the first-order transitions [13,14], where the probability of a song unit occurring depends solely on the previous song unit. More recent studies show that sequential dependencies can extend over long ranges beyond the previous song unit [10], and can be organized hierarchically across acoustic timescales (e.g. notes, motifs, phrases, bouts, songs) [11,15].

Apart from syntactic structure, recent interests in the temporal dynamics of birdsong have given rise to a rhythmic framework that enhances our understanding of birdsong complexity [16–19]. Song rhythm, an emergent property of how song units are timed in relation to one another [20], is a salient cue to songbirds [21,22]. In particular, some songbird species exhibit categorical rhythms, in which the ratios of consecutive intervals between song unit onsets fall into distinct categories [16,17], similar to how human music organizes rhythm [23]. Birdsong rhythm categories often include isochronous rhythms formed from roughly equal consecutive intervals [16,17], which are important to human music and speech [24]. Because birdsong and music share functions of attraction, existing theories on how music attracts listeners may be applicable to understanding how birdsong attracts songbirds [25].

Both syntactic and rhythmic frameworks contribute distinct insights to song complexity, and are typically studied separately. When investigating syntax, song rhythm is discarded when segmenting the song into a sequence of arbitrarily labelled units. Likewise, song rhythm analyses ignore symbolic labels and their sequential relationships. Yet, syntactic and rhythmic structure is imparted simultaneously to song. While it may be that these two feature spaces are independent and do not interact, we suggest a more likely possibility is that syntax and rhythm share a close relationship and work together to provide acoustic structure in communication signals. Thus, we argue for a novel approach to song complexity that combines both theoretical frameworks.

**Figure 1.**
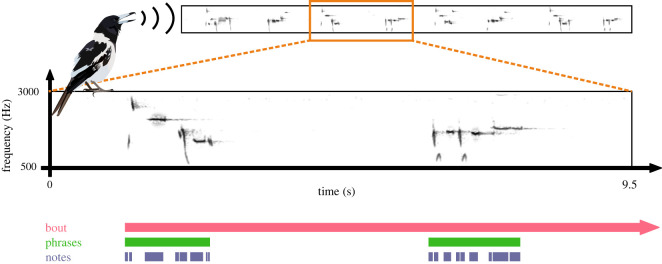
Pied butcherbird nocturnal formal song hierarchy. A song bout may span several hours. During a bout, short bursts of singing followed by periods of silence are characterized as phrases, and the individual spectro-temporal events that populate each phrase are characterized as notes.

Delivered nocturnally in the spring by a single bird, the formal songs of the Australian pied butcherbird (*Cracticus nigrogularis*) are particularly suitable for combined analyses of syntax and rhythm. Pied butcherbird formal songs are hierarchically organized into phrases, which are constituted by a sequence of spectro-temporally diverse notes (figure 1). At the note level, these formal songs are syntactically complex; notes are flexibly rearranged in different syntactic contexts [26], and even stereotyped note groups balance repetition and novelty, an attribute also familiar in human music [27]. The rhythmic structures of formal songs at the note level are correspondingly dynamic and diverse, and are similar to rhythmic devices described by music theory [26]. Additionally, formal songs are performed with immediate variety (where syntactic transitions occur between individual notes) [28], which allows for comparable analysis between syntactic and rhythmic features at the note level.

In this study, we investigated the statistical relationship between song syntax and rhythm in Australian pied butcherbird formal songs. We first defined song rhythm as the relative timing pattern of notes and analysed rhythm across the formal songs of three geographically diverse individuals with unique repertoires. We then examined the predictive relationship between the first-order sequential syntax and the analysed rhythm. We reveal that song syntax and rhythm are non-trivially related and offer interpretations of its possible cognitive functions and biomechanical origins.

## 2. Results

### 2.1. Pied butcherbird song is categorically organized

**Figure 2.**
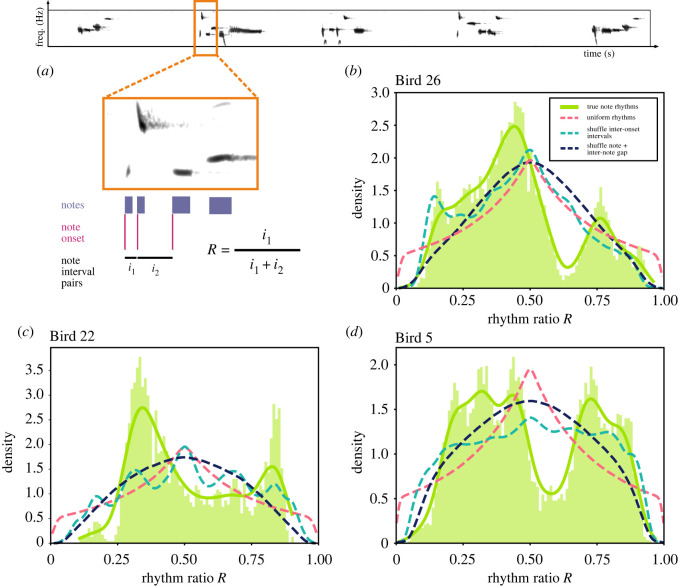
Pied butcherbird song rhythms are categorically organized. (*a*) Calculation of rhythm ratio *R* from note onsets and offsets. (*b*) Probability distribution of rhythm ratios for Bird 26. Rhythm ratios are pooled across all note types of the bird. The pink dashed line represents a uniform rhythm distribution, which assumes that song rhythm is generated from uniformly sampled inter-onset intervals. The teal dashed line represents a shuffled rhythm distribution where the order of inter-onset intervals in the song is shuffled. The dark blue dashed line represents a shuffled rhythm distribution where the order of notes and inter-note gaps are independently shuffled. (*c*,*d*) Probability distribution of rhythm ratios for Birds 22 and 5, as in (*b*).

We analysed field-recorded, nocturnal, formal, solo songs of wild Australian pied butcherbirds. The solo songs were recorded from three identified individuals (Birds 26, 22 and 5) that reside in different geographical locations and have unique repertoires (see §4.1). Given that all subjects have unique repertoires, we analysed each bird separately, and analysis data for each individual bird are pooled across the bird’s available recordings. To analyse the rhythmic structure of the songs, we manually segmented the songs at the note level, with each note given both an onset and an offset time (see §4.2). We extracted inter-onset intervals across the songs and estimated song rhythm *R* as the ratio between each interval and its combined length with the subsequent interval [17] (figure 2*a*; also see §4.3). Inter-onset intervals that extended across phrases were ignored, as their timescales were not comparable with inter-onset intervals within a phrase.

Over all three individual birds, we observed that pied butcherbird song rhythms were categorically organized at the note level. The distribution of song rhythms shows that some rhythm ratios are over-produced relative to chance and form discrete clusters, while other rhythm ratios are produced much more rarely than expected by chance (figure 2*b*–*d*). In comparison to a control distribution generated under the assumption that inter-onset intervals are uniformly distributed, the empirical distribution of song rhythm is considerably more clustered. To quantify the degree to which the rhythm ratios cluster, we calculated the Hopkins statistic (*H*_*B*26_ = 0.926, *H*_*B*22_ = 0.849, *H*_*B*5_ = 0.932) across the distribution of rhythm ratios, where *H* = 0 indicates similarity to a uniform distribution, *H* = 0.5 indicates similarity to a uniformly random distribution under a Poisson process, and *H* = 1 indicates highly clustered distribution (see §4.4). The rhythm ratio distributions for all three birds are highly clustered and unique.

**Figure 3.**
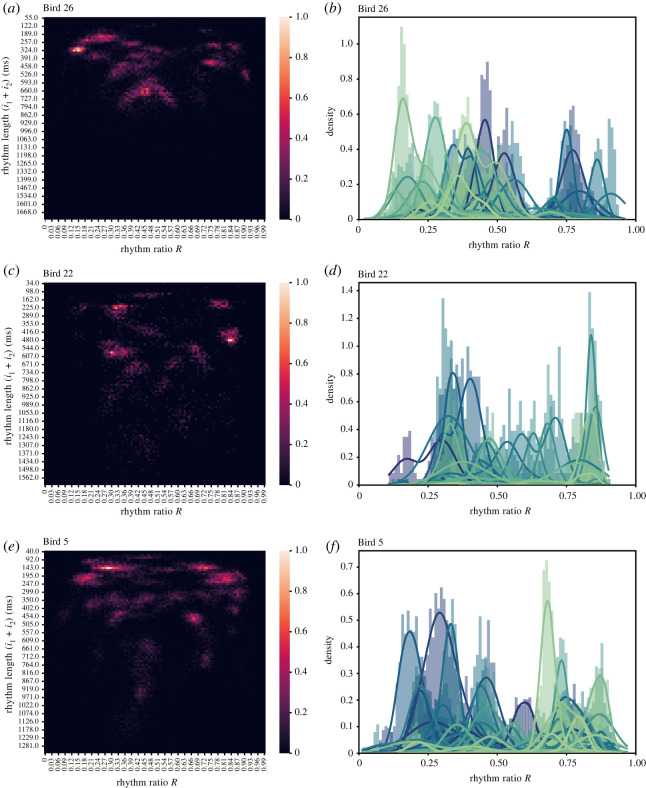
Categorical rhythms do not generalize across note types or rhythm lengths. (*a*,*c*,*e*) Heatmap showing the probability distribution of rhythms over various rhythm lengths for Birds 26, 22, 5. Rhythm length represents the combined length of the inter-onset intervals that constitute the rhythm ratio. (*b*,*d*,*f*) Rhythm distributions of unique note types for Birds 26, 22, 5. Each distinctly coloured portions of the histogram reflect rhythm ratios of a unique note type, where the unique note type is the first note in rhythm ratio calculation.

Rhythm clusters in human music and some animal vocalizations are generalized across tempo [17,29]. The tempo of a rhythm can be approximated as its rhythm length, which is the combined duration of a rhythm ratio’s constituent inter-onset intervals. Although the analysed pied butcherbird songs contain discrete rhythm clusters, such clusters minimally persist across a wide range of rhythm lengths (figure 3*a*,*c*,*e*). The central tendency of some rhythm clusters also drifts across rhythm lengths, suggesting that song tempo modulates how rhythm categories are presented in the song. We conclude that the pied butcherbird rhythm categories are variant to tempo.

It is also possible that pied butcherbird rhythm categories generalize across different note types. We used an established method [30] to first produce a symbolic sequence of computationally annotated pied butcherbird song notes defined by their onsets and offsets (see §4.5). The annotations were then manually reviewed and revised for accuracy. By visualizing the probability density of rhythm across unique note types, any rhythm categories that are shared across note types should have the same rhythm probability density. Across all three analysed birds, we observe no note types that share rhythm probability density (figure 3*b*,*d*,*f*), and conclude that rhythm categories in the analysed songs are note type dependent.

As rhythm categories in the analysed songs are note type dependent, they may be dependent on the song’s sequential structure as well. To broadly test the contribution of the song’s sequential structure to categorical rhythms, we created synthesized songs that shuffled the order of inter-onset intervals and compared such songs’ rhythm to the natural songs. Across all three birds, the rhythms of natural songs significantly differed from rhythms of shuffled songs (2-sample Kolmogorov–Smirnov test, *D*_*B*26_(8012, 8012000) = 0.110, *p*_*B*26_ < 0.001, *D*_*B*22_(2305, 2305000) = 0.112, *p*_*B*22_ < 0.001, *D*_*B*5_(13789, 13789000) = 0.083, *p*_*B*5_ < 0.001) (figure 2*b*–*d*), indicating that song rhythm modulates to the song’s sequential structure. As an inter-onset interval can be further broken down into a note and an inter-note gap, we also synthesized songs that shuffled both components independently. Similar to songs with shuffled inter-onset intervals, the rhythms of natural songs across all three birds significantly differed from rhythms of songs with shuffled notes and inter-note gaps (2-sample Kolmogorov–Smirnov test, *D*_*B*26_(8012, 9438000) = 0.174, *p*_*B*26_ < 0.001, *D*_*B*22_(2305, 2921000) = 0.107, *p*_*B*22_ < 0.001, *D*_*B*5_(13789, 15572000) = 0.095, *p*_*B*5_ < 0.001) (figure 2*b*–*d*). Overall, the sequential structures in the analysed songs significantly contribute to song rhythms.

### 2.2. The first-order sequential syntax predicts song rhythms

**Figure 4.**
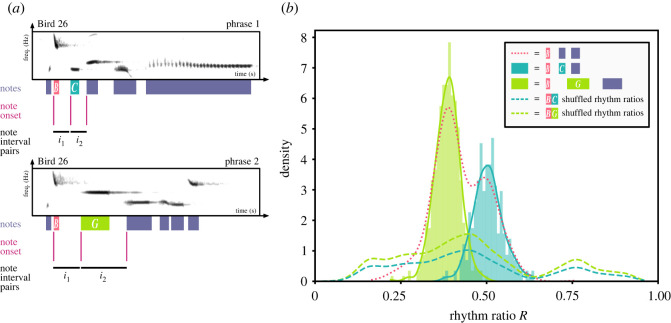
The first-order transitions predict note rhythm ratios. (*a*) Example of consistent first-order transitions from one note to two or more notes in an extended song of Bird 26. Within the entire song, note *B* consistently transitions to both note *C* (*p* = 0.345) and note *G* (*p* = 0.513). (*b*) Example of rhythm ratio distribution that is predicted by first-order transitions from note type *B* to note types *C* and *G*. The rhythm ratio distributions are coloured according to the first-order transition that generated the prediction. The dotted line represents rhythms predicted by a specific note type. Dashed lines represent shuffle-bootstrapped rhythms predicted by first-order syntax.

To further investigate the statistical relationship between song syntax and rhythm, we examined how components of song syntax affect rhythm. As pied butcherbird notes can often exist in different syntactic contexts [26], consistent first-order transitions (in which the probability of a note occurring is dependent on only the previous note) from one note to two or more notes are of particular interest to analyse. We found the subset of notes that have two or more consistent first-order transitions (see §4.6) and then used their first-order transitions as syntactic features of interest to compare with the rhythmic structure of the song. Note types that occur in fewer than 1% of rhythm ratios of the analysed individual are not included in the analysis. In total, we find 18 unique note types that have two or more consistent first-order transitions, with 38 first-order transitions in total (table 1). We tested if rhythm ratios are well predicted by such first-order transitions (figure 4*a*).

**Table 1.** 2-sample Kolmogorov–Smirnov test results for each analysed note type.

bird ID	note type	first-order transitions	transition probability	*R* count (*n*, *m*)^a^	${D_{n,m}}^{b}$	Bonferroni-corrected *p*
Bird 26	2 (*B*)	0 (*C*), 21 (*G*)	0.345, 0.513	276, 410	0.884	<0.001***
Bird 26	9	23, 33	0.443, 0.374	178, 160	0.255	<0.001***
Bird 26	15	9, 33	0.616, 0.275	246, 116	0.991	<0.001***
Bird 26	21	15, 26	0.287, 0.606	121, 255	1.000	<0.001***
Bird 26	24	28, 32	0.296, 0.640	148, 320	0.591	<0.001***
Bird 26	32	8, 10	0.598, 0.334	229, 128	0.356	<0.001***
Bird 22	6	15, 38	0.317, 0.409	59, 75	0.987	<0.001***
Bird 22	16	13, 25	0.429, 0.527	39, 48	0.752	<0.001***
Bird 22	17	26, 28	0.271, 0.581	34, 75	0.134	1.000
Bird 22	21	23, 29	0.370, 0.242	100, 66	0.975	<0.001***
Bird 22	23	2, 17	0.289, 0.383	90, 117	0.265	0.016
Bird 22	23	2, 27	0.289, 0.084	90, 25	1.000	<0.001***
Bird 22	23	2, 36	0.289, 0.103	90, 32	1.000	<0.001***
Bird 22	23	17, 27	0.383, 0.084	117, 25	1.000	<0.001***
Bird 22	23	17, 36	0.383, 0.103	117, 32	1.000	<0.001***
Bird 22	23	27, 36	0.084, 0.103	25, 32	0.145	1.000
Bird 22	24	19, 24	0.274, 0.406	26, 70	0.587	<0.001***
Bird 22	28	6, 23	0.465, 0.516	69, 82	0.131	1.000
Bird 22	38	16, 38	0.260, 0.212	87, 47	1.000	<0.001***
Bird 5	23	49, 59	0.282, 0.277	228, 223	0.996	<0.001***
Bird 5	49	34, 51	0.404, 0.303	291, 236	0.355	<0.001***
Bird 5	52	3, 37	0.356, 0.321	257, 225	0.333	<0.001***
Bird 5	58	43, 46	0.191, 0.275	248, 355	0.241	<0.001***

^a^Total rhythm ratio count for the two first-order transitions listed for each note type. *n* and *m* represent the rhythm ratio count of each first-order transition, respectively.

^b^2-sample Kolmogorov–Smirnov test statistic; also see §4.7.

Comparing the rhythm ratios associated with each first-order transition, we show that first-order transitions significantly predict song rhythm. When a set of rhythm ratios are predicted by only note type, the resulting rhythm ratio distribution can be multi-modal and contains discrete clusters (figure 4*b*). Identifying each rhythm by its first-order transition, however, reveals separate sub-distributions that clearly correspond to specific first-order sequences of notes (figure 4*b*). To verify our visual observations, we conducted 2-sample Kolmogorov–Smirnov tests for each analysed note type, with each sample consisting of the rhythm ratio distributions predicted by the note type’s first-order transitions (see §4.7). We then Bonferroni-corrected each *p*-value of all pairwise comparisons within a single bird to a family-wise error rate of *α* = 0.05. For 16 note types and 34 transitions analysed across three birds, the rhythm ratio distributions predicted by different first-order transitions are significantly different from each other (table 1; electronic supplementary material, figures S1–S4).

To verify that the first-order syntax predicts rhythms overall, we computed proportionate reduction of error (PRE) in song rhythms when predicted by all unique first-order transitions, and compared it to 95% shuffle-bootstrapped confidence intervals (see §§4.8 and 4.9). PRE quantifies how well one variable predicts another variable and is calculated as the proportion of variance that is reduced by the predictor variable. Overall, we show that the PRE of rhythm ratios is significantly high when predicted by sequential syntax across all observed first-order transitions (PRE_*B*26_ = 0.955; 95% CI_*B*26,shuffle-bootstrapped_ [0.030, 0.031], PRE_*B*22_ = 0.949; 95% CI_*B*22,shuffle-bootstrapped_ [0.065, 0.067], PRE_*B*5_ = 0.957; 95% CI_*B5*,shuffle-bootstrapped_ [0.054, 0.054]), where a PRE of 1 indicates full reduction in error, and a PRE of 0 indicates no reduction in error. This demonstrates that first-order transitions between pied butcherbird notes significantly predict rhythm ratios in the song overall.

### 2.3. Biomechanical influences in rhythm generation

All vocal communication is constrained by the physical manner in which vocalizations are produced. In songbirds, vocal production is primarily driven by the syrinx during exhalation [31], which imposes physical constraints associated with the production of certain spectro-temporal features such as pitch contour [32]. Song rhythm, similarly, may be subject to respiratory restraints on different note types. Assuming that pied butcherbirds take breaths between notes (see §3.3 for further discussion), certain notes may be physically harder to produce than others and subsequently require longer inhalation prior to production. In this sense, rhythm may be a biomechanical artefact of producing a sequence of song units that each have different respiratory demands. If true, then a clear relationship should be observed, such that longer notes are preceded by gaps that are, on average, longer than the gap that follows the note.

**Figure 5.**
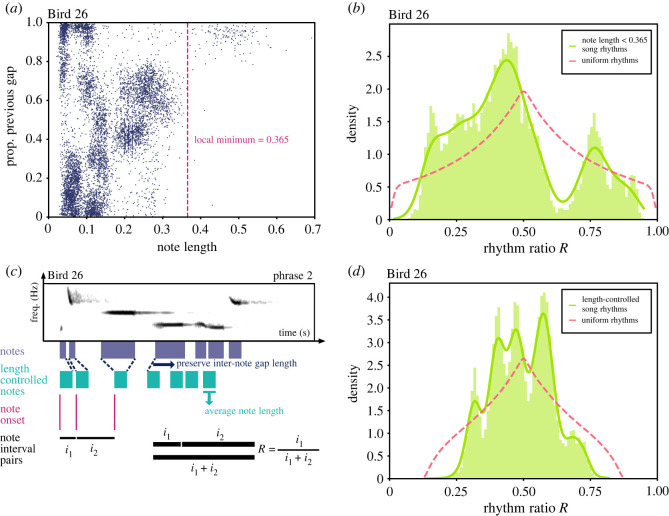
Song rhythm is likely not a biomechanical artefact of sequential song production. (*a*) The relationship between note length and proportion of previous inter-note gap relative to the total inter-note gap surrounding the note. A local note length density minimum is represented in dashed lines. (*b*) Rhythm ratio distribution of rhythms generated from notes less than a local minimum. (*c*) Calculation of a length-controlled rhythm ratio distribution. Note length is controlled to the average note length of the bout, and the inter-note gaps are unchanged. (*d*) Rhythm ratio distribution of length-controlled note rhythms.

In contrast to our prediction, no clear relationship between note length and the surrounding gap durations exists (figure 5*a*; electronic supplementary material, figure S5). Instead, notes across a wide range of lengths are associated with a wide range of gap durations. For notes spanning the great majority of observed lengths (Bird 26: length < 0.365 s; Bird 22: length < 0.450 s; Bird 5: length <0.500 s), durations of the surrounding gaps cluster as expected based on our previous results (figure 2*b*), but are broadly distributed around the centre point where the pre- and post-note gaps are of equal duration (ratio = 0.5;, figure 5*a*). The longest notes (Bird 26: length > 0.365 s; Bird 22: length > 0.450 s; Bird 5: length > 0.500 s) do appear to be strongly associated with longer pre-note gaps, but these make up a small proportion ($p_{B26}=2.57\text{\%}$, $p_{B22}=5.29\text{\%}$, $p_{B5}=0.74\text{\%}$) of the total notes. To test the contribution of these longer notes directly, we removed all rhythms that include notes longer than the above-stated thresholds and recomputed the distribution of rhythm ratios (figure 5*b*; electronic supplementary material, figure S5). The rhythm ratios changed minimally and remain categorically structured (*H*_*B*26,note length<0.365_ = 0.929, *H*_*B*22,note length<0.450_ = 0.849, *H*_*B*5,note length<0.500_ = 0.933). Applying a progressively stricter exclusion criterion at all local density minima in note length fails to abolish the categorical rhythm structure in the song (see electronic supplementary material, figures S6–S8).

As an additional control for any effects of note length on rhythm, we calculated controlled rhythm ratios where all note lengths were artificially set to the average note length for each bird (figure 5*c*), then recomputed the rhythm ratio distribution. Again, rhythms remain categorically organized (*H*_*B*26length-controlled_ = 0.983, *H*_*B*22length-controlled_ = 0.932, *H*_*B*5length-controlled_ = 0.989; figure 5*d*; electronic supplementary material, figure S5). Likewise, in note-length-controlled songs, the PRE in song rhythm predicted from the first-order note transitions remains significantly elevated above the null distribution (PRE_*B*26_ = 0.909, 95% CI_*B*26,shuffle-bootstrapped_[0.030, 0.031], PRE_*B*22_ = 0.775, 95% CI_*B*22,shuffle-bootstrapped_[0.066, 0.068], PRE_*B*5_ = 0.909, 95% CI_*B5*,shuffle-bootstrapped_[0.053, 0.054]). The note rhythm structure and its interaction with syntax are not artefacts of patterned differences in note length.

### 2.4. Long-range dependencies in pied butcherbird song

In our previous analyses, we examined the relationship between first-order sequential syntax and rhythm ratios at the note level. Such analyses were limited by the lack of considerations for sequential syntactic complexities beyond what can be described by a first-order Markov model. It is known, however, that other songbird species show long-range sequential dependencies within their song structure [10,11,33], and the interaction between syntax and rhythm may well extend beyond the timescale we have analysed. Here, we demonstrate that pied butcherbird song syntax and rhythms may also exhibit long-range sequential structures.

#### 2.4.1. Long-range order in syntax

**Figure 6.**
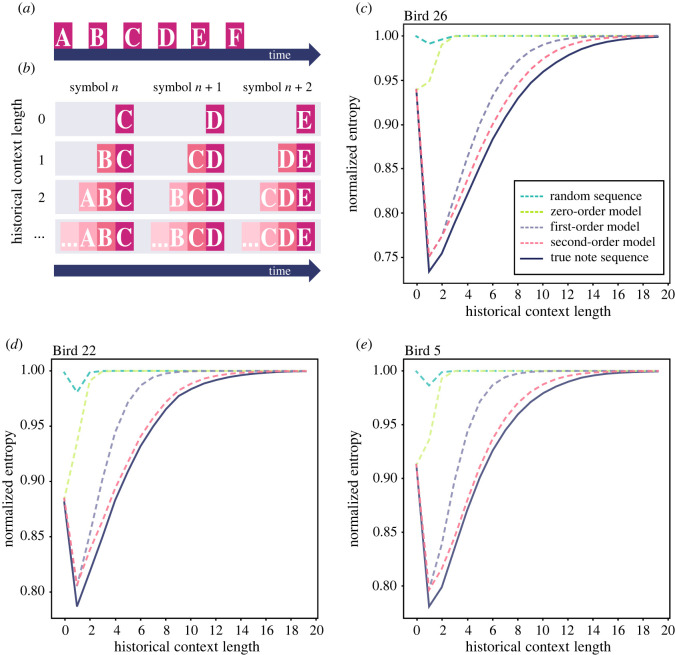
Note sequence exhibits long-range sequential dependencies. (*a*) Example of an observed note sequence. (*b*) Diagram illustrating how sequences with different historical context lengths are generated. At historical context length 0, the normalized entropy is calculated over the true note sequence. At historical context lengths above 0, the normalized entropy is calculated over symbols representing the combination of the currently observed note and a number of its previous notes up to the historical context length. For example, for historical context of 1, the sequence is constructed such that each symbol of the sequence is a combination of the currently observed note and its previous note. For historical context of 2, the sequence is constructed such that each symbol of the sequence is a combination of the currently observed note and its two previous notes, etc. (*c*) The normalized entropy of the true observed note sequence is consistently lower than all other simulated sequences across historical context lengths, including its second-order model. (*d*,*e*) Normalized entropy analyses for Birds 22 and 5.

To test for syntactic structures more complex than the first-order sequential dependencies, we synthesized song sequences using Markov models (zeroth, first and second order) and compared the temporal structure of synthesized song sequences to the true song sequence. To estimate the long-range information structure of song sequences, we constructed additional versions of the same song sequence that take into account previously sung notes (historical context) for each song sequence (true or synthesized). In the original version of each song sequence where no historical context is considered, each symbol in the sequence represents each note (figure 6*a*). In additional versions, each symbol in the sequence represents the combination of each note and some number of its previous notes. The number of previous notes added to each note in the sequence is the historical context length of the note sequence (figure 6*b*). We estimate long-range information structure as the normalized entropy over song sequences across different historical contexts (see §4.10). If the true note sequence contains long-range sequential structures more complex than the first-order sequential dependencies, it should have lower normalized entropy than note sequences synthesized by a first-order Markov model.

In comparing the normalized entropy of the true note sequence to synthesized note sequences, we show that the normalized entropy of the true note sequence is consistently lower than synthesized sequences across different historical contexts (figure 6*c*–*e*). Specifically, the normalized entropy of the true note sequence (*M*_*B*26_ = 0.921, *M*_*B*22_ = 0.945, *M*_*B*5_ = 0.942) is lower than note sequences synthesized from a first-order Markov model (*M*_*B*26_ = 0.943, 95% CI_*B*26_ [0.943, 0.944], *M*_*B*22_ = 0.967, 95% CI_*B*22_ [0.967, 0.967], *M*_*B*5_ = 0.967, 95% CI_*B*5_ [0.967, 0.967]) across historical context lengths, suggesting the presence of long-range sequential dependencies in pied butcherbird note sequences. Furthermore, the normalized entropy of the true note sequence (*M*_*B*26_ = 0.921, *M*_*B*22_ = 0.945, *M*_*B*5_ = 0.942) is lower than note sequences synthesized from a second-order Markov model (*M*_*B*26_ = 0.932, 95% CI_*B*26_ [0.931, 0.932], *M*_*B*22_ = 0.951, 95% CI_*B*22_ [0.951, 0.952], *M*_*B*5_ = 0.949, 95% CI_*B*5_ [0.949, 0.949]), which is a long-range sequential model in which the probability of a song unit occurring is dependent on the previous two song units. Thus, analysed pied butcherbird note sequences contain long-range structure beyond what is explained by the first- and the second-order Markov models.

#### 2.4.2. Phrase-level structured rhythm

Given our observation of long-range dependencies in pied butcherbird songs, we sought to examine rhythm at longer timescales. Pied butcherbird phrases in the analysed dataset are produced at a significantly larger timescale than notes, where the mean inter-onset interval for phrases is around 7.449 s (95% CI [6.876, 8.022]) and the mean inter-onset interval for notes is around 0.199 s (95% CI [0.197, 0.200]). Analysing pied butcherbird phrase rhythms, therefore, allows us to examine long-range rhythmic dependencies beyond the note level.

**Figure 7.**
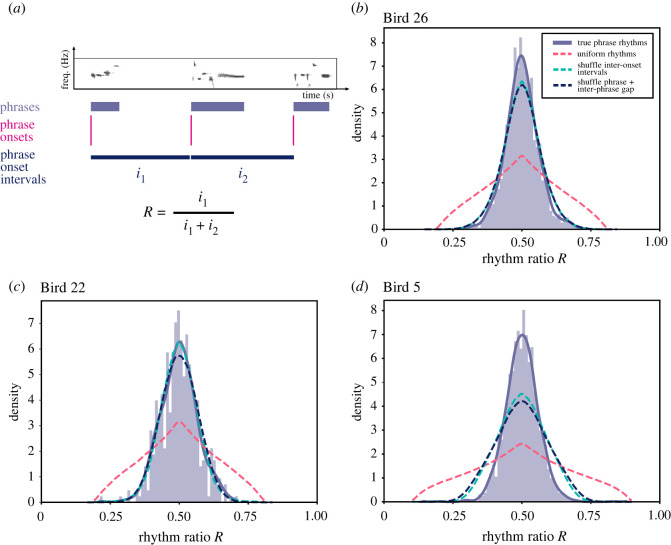
Inter-phrase rhythms are isochronously organized. (*a*) A diagram demonstrating the calculation of inter-phrase rhythm ratios. (*b*) Rhythm ratio distribution of inter-phrase rhythms over the entire recording for Bird 26. Uniform phrase rhythms are plotted in dashed pink lines. Phrase rhythms with shuffled inter-onset intervals are plotted in teal lines. Phrase rhythms with independently shuffled phrases and inter-phrase gaps are plotted in dark blue lines. (*c*,*d*) Inter-phrase rhythms for Birds 22 and 5, as in (*b*).

To calculate phrase-level rhythms, we denoted phrase onsets as the onset of the first note in the phrase and calculated inter-phrase onset intervals over the entire song. Similar to note rhythms, we estimated the phrase rhythm ratio as the ratio between an interval and its combined length with the subsequent interval (figure 7*a*).

In contrast with notes, we observe that inter-phrase rhythms are isochronously organized, showing that structured patterns of song rhythm exist beyond note level, but are less varied. The inter-phrase rhythm ratios of the song cluster around 0.5, indicating that consecutive phrase onset intervals are similar in length to each other (figure 7*b*–*d*). When compared to shuffle-bootstrapped rhythm distributions where the ordering of phrase onset intervals is randomized, true phrase rhythms of two birds cluster more closely around a rhythm ratio of 0.5 (2-sample Kolmogorov–Smirnov test, *D*_*B*26_(1385, 1385000) = 0.050, *p*_*B*26_ = 0.002, *D*_*B*5_(1607, 1607000) = 0.111, *p*_*B*5_ < 0.001) while Bird 22 showed no difference (2-sample Kolmogorov–Smirnov test, *D*_*B*22_(426, 426000) = 0.029, *p*_*B*22_ = 0.865). Similar results arise in comparing songs that shuffle phrase duration and inter-phrase gap independently (2-sample Kolmogorov–Smirnov test, *D*_*B*26_(1385, 1410000) = 0.054, *p*_*B*26_ < 0.001, *D*_*B*5_(1607, 1688000) = 0.125, *p*_*B*5_ < 0.001, *D*_*B*22_(426, 533000) = 0.035, *p*_*B*22_ = 0.665). This suggests that some pied butcherbirds may actively maintain a more isochronous rhythm at the phrase level of song organization, but not others. Together with the observation of categorical rhythms at the note level, these results provide preliminary evidence that pied butcherbirds can maintain rhythm structure across multiple levels of song structure and timescales.

## 3. Discussion

This study combines two analysis frameworks to examine the rhythmic and syntactic structure of a natural communication signal. Each of these frameworks seeks to understand the temporal complexity of the signal, but they are normally applied independently. We show that the songs of wild Australian pied butcherbirds in our sample are organized both rhythmically and syntactically, and that rhythm and syntax interact with each other. The rhythmic structures of these songs are specific to each singer, and the song’s rhythms are reliably predicted by its first-order sequential syntax. The observed relationships between syntax and rhythm remain significant after controlling for note length, indicating this temporal complexity does not emerge from trivial biomechanical constraints. The strong relationship between syntax and rhythm in pied butcherbird songs prompts further questions regarding its ethological function, the biomechanical origins, and the prevalence of similar interactions in other species’ vocal signals.

### 3.1. Interaction of syntax and rhythm

#### 3.1.1. Syntactic–rhythmic relations in birdsong

The interaction between syntax and rhythm has, to our knowledge, only been addressed in one previous study of birdsong. Results from this prior work, where song rhythm was quantified using the distribution of inter-onset intervals [34], show that Bengalese finch songs with more discrete clusters of inter-onset intervals have more unique note types, but are not more unique transitions between notes. This suggests that in this species unique inter-note gaps are associated with each note type, but not necessarily tied to syntactic variation. One reason these results differ from ours may be due to species differences between the spectro-temporal structures of pied butcherbird and Bengalese finch songs. Unlike Bengalese finch songs, pied butcherbird songs do not exhibit serial redundancy, whereby a vocal unit is repeated consecutively before transitioning to a new unit which is thought to counter signal loss in noisy communication environments [35]. Although pied butcherbird songs exhibit strong stereotypy in their sequential organization [26], they are delivered with immediate variety as opposed to serial redundancy. In the absence of serial redundancy, it may be that pied butcherbirds use syntax and rhythm to aid temporal predictability and avoid information loss due to noise-induced signal degradation or corruption. In this sense, various solutions to maintain signal fidelity for the benefit of the receiver may generate selection pressures that shape song complexity. In the case of pied butcherbirds, this may yield more varied control of song rhythm. Changes in rhythm categories may be especially salient cues that capitalize on general predictive coding principles [36] and reinforce syntactic encoding, as discretized rhythms may be easier to learn and transmit [35].

A second distinction between prior work [34] and the current study is purely methodological. Instead of characterizing birdsong rhythm by the distribution of single inter-onset intervals [34], we chose to quantify the relative timing between consecutive inter-onset intervals under the assumption that rhythm is an emergent temporal pattern that minimally requires two or more consecutive intervals. Given that songbirds are capable of flexibly perceiving rhythms independent of absolute timing information [21,22], a relative-timing approach is likely to produce findings that are more behaviourally relevant. Another advantage to our relative-timing approach is that it emphasizes how rhythm contributes to a song’s dynamic structure over time. We suggest it is likely that the relational change in rhythmic structure over time modulates listener expectation, which is hypothesized to be a contributing factor in birdsong attractiveness [25].

#### 3.1.2. Syntactic–rhythmic relations are important in human speech and music

Although different in important ways, birdsong shares many similar acoustic properties and neural correlates to human speech [11,37] and music [17,38]. It may, therefore, be useful to understand the implications of syntactic–rhythmic interactions in birdsong from a comparative perspective. Interactions between syntax and rhythm play important roles in the perception of both human speech and music. Specifically, unexpected rhythms in speech and music have been shown to modulate linguistic and musical syntax processing [39], and it is hypothesized that such disruptions are due to shared cognitive resources between linguistic syntactic and musical rhythmic processing [40]. In support of this shared resource hypothesis, normally developing children that show better rhythm discrimination also performed generally better on a grammar test [41]. Likewise, in children with developmental language disorders, external temporally-regular rhythmic stimulation improves judgments of grammatical syntax [42]. Most interestingly, the event-related potential of metrical rhythm processing and syntactic processing have degrees of overlap, suggesting a neural response that may correlate to the integration of rhythmic and syntactic information [43].

Interactions between syntax and rhythm also appear in computational models of musical syntax. For example, an analysis of folk song melodies showed that syntactic units with stronger rhythmic functions (metrically stressed rhythms or final rhythms) tend to persist (show fewer changes) over cultural iterations [44], suggesting that certain rhythms can increase the salience of syntactic sequences. Rhythm also facilitates syntactic encoding on a computational level in music processing. A combined model of musical harmony, incorporating both harmonic syntax and rhythmic information, outperforms other models with only syntax or rhythmic information in predicting the latent structure of musical sequences [45]. Similar improvements in sequence prediction over productive and perceptual models that only include syntactic encoding may be feasible for birdsong as well. Future experiments that investigate the perception and production of rhythm-modulated syntactic song sequences in songbirds will be important to understanding the comparative bases of underlying cognitive processes and mechanisms.

### 3.2. Timescale of interaction between syntax and rhythm

The interaction between syntax and rhythm we show in pied butcherbird song is limited to a single timescale encompassing consecutive inter-onset intervals and the first-order note transitions. Both syntactic and rhythmic features can, however, extend over much longer timescales. Long-range sequential dependencies between song elements, extending well beyond those captured by the first-order models, have been observed for several songbird species [10,11]. Our present results show that pied butcherbird songs in our sample also exhibit long-range dependencies beyond the first order. The upper bound and precise form of these dependencies are unknown, as is the degree to which song rhythm interacts with longer-range sequential dependencies and syntax. Here again, expectations induced and maintained by rhythmic regularities may help to emphasize specific syntactic events and/or facilitate the memorization of syntactic sequences [17,42,46]. Our observations of isochronous rhythms at the phrase level in select pied butcherbird songs suggest this as an important timescale for future work to search for interactions between syntax and rhythm. Owing to the highly variable nature of pied butcherbird phrases caused by syntactic flexibility at the note level [26], a syntactic analysis at the phrase level requires a sample size beyond what we are reasonably able to analyse. Finally, we note that the interaction between syntax and rhythm can be explored at elastic timescales using modern computational techniques. There has been rising interest in modelling birdsong syntax with latent space trajectories, which affords spectro-temporal detail at scales below the level of discretized units [30]. Similarly, rhythmic features of syntax can also be analysed on a much smaller scale, such as by calculating the Allan factor variance [47] or multifractality [18] of the signal amplitude envelope. Such approaches to modelling syntax and rhythm have the advantage of bypassing assumptions of timescale and element discretization and may be fruitful to investigate in the future.

### 3.3. Syntactic–rhythmic relations in the context of biomechanical constraints

Animal vocalizations are subject to their physical means of production, and in the limit, the production of birdsong syntax and rhythm must be constrained by physiology. Birdsong is predominantly produced via exhalation, and its spectro-temporal structure strongly reflects the respiratory and muscular constraints imposed by the syrinx and the syringeal muscles [31,32]. Although we did not examine the actual physical vocal production of the pied butcherbird, we searched for potential effects of biomechanical constraints by estimating the effects of respiration and note length on syntactic–rhythmic relations. We show evidence that note length does not significantly impact the song’s rhythmic structure, and that controlling for note length does not remove syntactic–rhythmic relations in the song. Together, this suggests that song rhythm is not likely a biomechanical artefact of respiration needs or patterned variations in note length, and that syntactic–rhythmic relations in pied butcherbird songs do not result from respiratory constraints of sequential vocal production.

We did, however, observe a small but reliable effect that may be tied to biomechanical influences on song rhythms and their relationship to syntax for the very longest of notes comprising about 0.74–5.29% of the note repertoire of each bird. Interestingly, the distribution of inter-note gap is most variable when note length is short, suggesting that motor constraints for short notes may be less than those of longer notes. It may be that because shorter notes have fewer motor constraints, they can more flexibly be incorporated into more variable rhythmic patterns.

Of course, other features of notes and different syntactic units, beyond simple note duration, likely impose stresses on a songbird’s vocal production capacities. For example, notes with higher frequency content or amplitude may require more subglottal pressure to produce [48] and be more physically constrained. Birdsong trills, in particular, can be generated in two ways, either with mini-breaths between trill elements or with a sustained breath [31], and are likely at the extreme of vocal ability. We presumed in our analyses that pied butcherbirds take breaths between notes, and that trill notes are produced with sustained breath, but that is not known. We also did not estimate the constraints imposed by syringeal muscles, but as they interact with respiration in song production, they may contribute to syntactic–rhythmic relations [31]. Further studies in birdsong mechanics may further elucidate the extent to which syntactic–rhythmic relations are influenced by songbird physiology.

### 3.4. Towards a systematic conception of song complexity

The complexity of birdsong has a rich but idiosyncratic history of investigation and is often described by seemingly independent song features tied to sexual selection, such as repertoire size, production rate, song familiarity or syntactic regularity [1,7]. Perhaps the most common view of song complexity is to consider it a function of repertoire size [49]. Although repertoire size is a reliable predictor of female preferences in some model species, a deeper look into the actual effect of repertoire size reveals limitations to this approach. Meta-analyses of repertoire size indicate that female preferences for large repertoires in model species are inconsistent between laboratory and field studies in many cases, and not widespread across songbird species [49,50]. Even among studies that report significant preferences for large repertoires, the effect is generally small [49]. One way to interpret these results is that a singular-feature approach to song complexity will likely address only a small portion of a signal’s function.

Here we demonstrated how two features of song complexity traditionally studied in isolation, song syntax and song rhythm, have meaningful interactions with each other. We theorize that the interactions between syntax and rhythm contribute to modulating listener expectation and may be critical to the signal’s function. Perhaps the most general conclusion to be drawn from our work is that song complexity may best be considered as an interactive and emergent property of the communication system. In this respect, it may be more important to study the relationships between song features than it is to study the features themselves.

## 4. Methods

### 4.1. Song recording

All 7 analysed pied butcherbird nocturnal formal songs were field-recorded on an Olympus LS-11 Linear PCM Field Recorder with a pair of Sennheiser ME67 shotgun microphones mounted on a tripod. Songbird identity, subspecies, location, time and recording information for all recordings is located in table 2. As pied butcherbirds are non-migratory, we provided tentative subspecies identification based on the geographical location of the recording sites [27,51]. Pied butcherbirds are sexually monomorphic and indistinguishable in the field [51], and the sex of the pied butcherbirds in the recordings is not identified. We presume that the subjects in our dataset did not vocally interact with other conspecifics based on field observations and subjective evaluation of recordings, but we do not rule out its possibility as minimal sections of recordings contain vocalization from non-subject conspecifics. For further details on the recordings, see electronic supplementary material, S1.

**Table 2.** Song recording information.

recording ID	bird ID	subspecies	location	time	sampling and bit-rate
110395	Bird 26	*Craticus nigrogularis picatus*	Alice Springs, Northern Territory on Ragonesi Road at Palm Place	28 July 2017 3.45	48 000 Hz, 24-bit
110593	Bird 22	*Craticus nigrogularis picatus*	Alice Springs, Northern Territory at the Araluen Arts Centre on Larapinta Drive	8 Sep 2018 3.45	48 000 Hz, 24-bit
110640	Bird 22	*Craticus nigrogularis picatus*	Alice Springs, Northern Territory at the Araluen Arts Centre on Larapinta Drive	1 Oct 2018 1.52	48 000 Hz, 24-bit
110131	Bird 5	*Craticus nigrogularis nigrogularis*	Georgetown, Queensland, at the pool in Greens Park	9 Oct 2015 4.45	44 100 Hz, 16-bit
110136	Bird 5	*Craticus nigrogularis nigrogularis*	Georgetown, Queensland, at the pool in Greens Park	10 Oct 2015 4.45	44 100 Hz, 16-bit
110143	Bird 5	*Craticus nigrogularis nigrogularis*	Georgetown, Queensland, at the pool in Greens Park	12 Oct 2015 4.25	44 100 Hz, 16-bit
110146A	Bird 5	*Craticus nigrogularis nigrogularis*	Georgetown, Queensland, at the pool in Greens Park	13 Oct 2015 4.30	44 100 Hz, 16-bit

All analysed song data for an individual bird are pooled across its available recordings, given that the repertoires of each bird are unique from one another but are consistent within an individual. Analyses for Bird 26 are performed on one extended bout in one recording, while analyses for Bird 22 are pooled across two recordings, and analyses for Bird 5 are pooled across four recordings.

#### 4.1.1. Digital audio de-noising

The pied butcherbird recordings were pre-processed for analysis in iZotope RX 8. The recordings were processed by a dynamic de-noising process that adaptively samples the local noise spectrum in the recording and subtracts it from the signal. Reverberation artefacts are also subtracted from the signal. As most of the features of pied butcherbird songs are contained within its fundamental frequency range of 0.5–3 kHz, the signal was bandpass filtered accordingly. For exact signal-processing settings, see electronic supplementary material, S4.

### 4.2. Song unit segmentation

The song units (phrases and notes) of the pied butcherbird songs are first segmented computationally by a dynamic-threshold-based automatic segmenter [52]. We define pied butcherbird notes as discontinuous instances of acoustic events surrounded by silence [53], and pied butcherbird phrases to be 1–3 s of continuous singing surrounded by silence [28]. The resulting textgrid segmentations are then manually reviewed and adjusted for accuracy in Praat; the segmentation boundaries for notes are checked with both the waveform amplitude and the spectrogram of the song, and the segmentation boundaries for phrases are aligned with the onset and offset of the first and last note, respectively, of each phrase. For more information on song unit segmentation, see electronic supplementary material, S2.

Spectrogram representations of each note and phrase segmentation are created by first normalizing the signal, filtering the waveform by the pied butcherbird species-specific frequency range (500–3000 Hz), and then performing one-sided short-time Fourier transformation with a window length of 9 ms and a hop length of 1 ms. Frequencies within generated spectrograms are then scaled with a Mel filterbank. The final generated spectrograms have 64 frequency bins scaled across the range of 500–3000 Hz. In our sample, we analysed seven bouts of song from three birds (Birds 26, 22, 5) comprising 3847 phrases and 31 767 notes. The distribution of segmented notes across all recordings can be seen in table 3.

**Table 3.** Song segmentation information.

recording ID	bird ID	recording length	cum. phrase length	phrases	cum. note length	notes
110395	Bird 26	02:04:16	00:39:41	1427	00:26:07	10 866
110593	Bird 22	00:59:17	00:11:55	431	00:07:19	2489
110640	Bird 22	02:04:16	00:04:32	218	00:02:53	1082
110131	Bird 5	01:06:17	00:13:47	452	00:09:04	4403
110136	Bird 5	01:03:30	00:10:09	373	00:06:26	3379
110143	Bird 5	00:59:52	00:13:52	457	00:08:53	4573
110146A	Bird 5	01:06:00	00:15:21	489	00:09:57	4975

### 4.3. Rhythm ratio calculation

We followed the methods proposed by Roeske *et al.* [17] to estimate song rhythm. Onsets of segmented pied butcherbird notes are first used to calculate inter-onset intervals *i*_*n*_ across the entire recording. The rhythm ratio *R* is then calculated as the ratio of every inter-onset interval against its combined length with the subsequent interval:

4.1 $$R=\frac{i_{n}}{i_{n}+i_{n+1}}.$$

To estimate the phrase level rhythm ratios *R* of pied butcherbird song, the inter-onset intervals are calculated from phrase onsets instead:

4.2 $$R=\frac{i_{\;p}}{i_{\;p}+i_{\;p+1}}.$$

### 4.4. Hopkins statistic

We used the Hopkins statistic to quantify the degree to which rhythm ratios are categorically organized. The Hopkins statistic is a common measure of data clusterability, in which the nearest-neighbour relation of the empirical data is compared with a random Poisson point process [54]. Highly clustered data will reflect a Hopkins statistic of 1, while Poisson processed random data and uniform data will reflect a Hopkins statistic of 0.5 and 0, respectively. To obtain a Hopkins statistic that is representative, we generated a sampling distribution of 100 Hopkins statistics and used the mean of the sampling distribution as an approximation of the dataset’s true Hopkins statistic:

4.3 $$\mathrm{Hopkins}\;\mathrm{statistic}=\frac{\sum_{i=1}^{m}u_{i}^{d}}{\sum_{i=1}^{m}u_{i}^{d}+\sum_{i=1}^{m}w_{i}^{d}}.$$

### 4.5. Semi-supervised note annotation

We used a combination of computational methods and manual correction to annotate the segmented notes. We first used the methods described by Sainburg *et al.* [30] to computationally project segmented spectrograms into a latent feature space with uniform approximation and projection (UMAP) [55]. UMAP first creates a graphical representation of the dataset by constructing a nearest-neighbour graph of high-dimensional data, and then embeds the data into a low-dimensional space in a way that preserves the structure of said nearest-neighbour graph. This transforms the original data into a latent feature space that has been shown to be useful in characterizing bioacoustic data [11,30,56].

To enhance the computational efficiency of UMAP, we linearly rescaled the spectrograms that are fitted by UMAP along their time dimension by a factor of 0.1 while preserving note-length variability in constructing the feature space. The spectrograms are then zero-padded to the maximum rescaled note length in the dataset. After the spectrograms are pre-processed, UMAP is fitted over the spectrograms (where each time–frequency bin is treated as a dimension) to construct a two-dimensional latent feature space that graphically characterizes the dataset. To obtain a latent feature space that retains global structures as much as possible without compromising local structure resolution, 1% of the total spectrograms is used in computing the nearest-neighbour graph, and the points are embedded in the low-dimensional space without a minimum distance between.

As UMAP clusters data into discrete categories, unsupervised latent data clustering methods can be employed to extract data categories out of a latent feature space. We employed hierarchical density-based spatial clustering of applications with noise (HDBSCAN) to annotate clusters in the UMAP latent feature space as discrete note and phrase types [57]. For each latent feature space generated, HDBSCAN builds a graphical hierarchy of data points using a minimum spanning tree, and then generates discrete labels for clusters that are larger than 1% of the total data point in the latent space. Data that HDBSCAN labels as noise are grouped with the nearest valid HDBSCAN label with Scikit-learn [58].

To derive more accurate note categories that avoid grouping of similar but discrete notes, we iteratively reprojected and relabelled each note category with UMAP and HDBSCAN, developing fine labels for each note category. This process is repeated recursively for each projection until only one cluster can be identified from the projection, with each recursion using progressively higher spectrogram resolution in the time dimension. This creates an excessive number of highly accurate note categories that can be manually grouped and curated with spectrographic and syntactic references, resulting in a largely accurate dataset (see electronic supplementary material, S3).

### 4.6. Parsing consistent first-order transitions

To filter consistent first-order transitions, we first modelled a bootstrapped zero-order sequence of note annotations that retains only the frequency of occurrence for each note type. The length of the bootstrapped zero-order note sequence is a hundred times the length of the observed note sequence extracted from the song. By calculating all the first-order transition probabilities for this bootstrapped zero-order note sequence, we obtained a null probability distribution of the first-order transitions. We define consistent first-order transitions as any empirical transitions that have transition probabilities higher than their 95% confidence intervals derived from the null probability distribution. Consistent first-order transitions must also appear an amount of times equal to 0.01 of the combined sequence length across bouts for each bird.

### 4.7. 2-sample Kolmogorov–Smirnov test

We used 2-sample Kolmogorov–Smirnov tests to evaluate the difference in rhythm distribution as predicted by the first-order syntax. This test evaluates whether or not two distributions can be derived from the same parent distribution. The Kolmogorov–Smirnov statistic is defined as

4.4 $$D_{n,m}=\sup|F_{1,n}(x)-F_{2,m}(x)|.$$

The null hypothesis is rejected if

4.5 $$D_{n,m}>\sqrt{-\ln\left(\frac{\alpha }{2}\right)\cdot \frac{1+(m/n)}{2m}}.$$

We specifically chose this statistical test since it does not assume the shape of the underlying distribution. This allows us to compare rhythm distributions without making assumptions of how pied butcherbirds generate rhythms.

For note types that have more than two consistent first-order transitions, pairwise 2-sample Kolmogorov–Smirnov tests across all combinations of the first-order transitions were conducted.

### 4.8. Bootstrapping rhythm ratio distribution with dissociated syntax

To simulate the null hypothesis in which pied butcherbird song rhythm ratios are not related to syntax, we generated shuffled song sequences where the song syntax and song rhythm ratio distribution are individually preserved, but the relationship between syntactic and timing information is disassociated. To obtain a robust shuffled sequence, we bootstrapped 100 shuffled sequences and used such sequences to generate null predictions of syntactic–rhythmic relations.

### 4.9. Proportionate reduction of error

PRE is a statistical test for evaluating the amount of variance explained by a model. We use PRE to evaluate the amount of variance in song rhythm predicted by the first-order transitions ($\hat{Y}$) in comparison to the amount of variance in song rhythm explained by the mean song rhythm ($\bar{Y}$):

4.6 $$\mathrm{PRE}=1-\frac{\sum {\left(Y_{i}-\hat{Y}\right)}^{2}}{\sum {\left(Y_{i}-\bar{Y}\right)}^{2}}.$$

### 4.10. Long-range sequential syntax

We adapted the methods of Markowitz *et al.* [10] to characterize long-range order in pied butcherbird songs. We first synthesized pied butcherbird note sequences with different sequential models, which include a random model that only preserves the amount of total note type, a zeroth-order Markov model that only preserves the amount and distribution of note types, a first-order Markov model that only preserves the transition probabilities dependent on the previous note, and a second-order Markov model that preserves the transition probabilities dependent on two previous notes. We synthesized 100 note sequences for each model. For each note sequence, we then created additional versions of the same note sequence where each note is considered with its previous notes (song history). In the original version of each note sequence where no historical context is considered, each symbol in the sequence represents each note. In additional versions of each note sequence, each symbol in the sequence represents each note combined with a number of its previous notes, and the number of its previous notes is the historical context length.

To calculate the amount of information structure in each song sequence, we used a standard formula for normalized entropy [59], where *n* represents the number of unique symbols in each sequence:

4.7 $$\eta (X)=-\sum_{i=1}^{n}\frac{\;p(x_{i})\log_{b}(p(x_{i}))}{\log_{b}(n)}.$$

We created additional note sequences of up to 20 historical contexts, as normalized entropy for all note sequences with more than 20 historical contexts shows minimal differences.

Normalized entropy can also be calculated where *n* only represents the number of unique notes in the empirical sequence without historical context. Designating *n* as the number of possible notes and their respective historical contexts in each sequence, however, allows us to draw the same inferences, as well as provides more information as to how the organization of the signal fluctuates across historical contexts.

## Data Availability

The Australian pied butcherbird dataset is available on Zenodo [60]. All analyses performed in this study are available at https://github.com/xingjeffrey/syntax_rhythm_pbb. The data are provided in electronic supplementary material [61].
